# Metabologenomics reveals strain-level genetic and chemical diversity of *Microcystis* secondary metabolism

**DOI:** 10.1128/msystems.00334-24

**Published:** 2024-06-25

**Authors:** Colleen E. Yancey, Lauren Hart, Sierra Hefferan, Osama G. Mohamed, Sean A. Newmister, Ashootosh Tripathi, David H. Sherman, Gregory J. Dick

**Affiliations:** 1 Department of Earth and Environmental Sciences, University of Michigan, Ann Arbor, Michigan, USA; 2 Program in Chemical Biology, University of Michigan, Ann Arbor, Michigan, USA; 3 Life Sciences Institute, University of Michigan, Ann Arbor, Michigan, USA; 4 Departments of Medicinal Chemistry, Chemistry, Microbiology, and Immunology, University of Michigan, Ann Arbor, Michigan, USA; 5 Natural Products Discovery Core, Life Sciences Institute, University of Michigan, Ann Arbor, Michigan, USA; 6 Pharmacognosy Department, Faculty of Pharmacy, Cairo University, Cairo, Egypt; 7 Cooperative Institute for Great Lakes Research (CIGLR), School for Environment and Sustainability, University of Michigan, Ann Arbor, Michigan, USA; Wageningen University, Wageningen, the Netherlands

**Keywords:** cyanoHABs, secondary metabolism, *Microcystis*, metabologenomics, natural products

## Abstract

*Microcystis* spp. are renowned for producing the hepatotoxin microcystin in freshwater cyanobacterial harmful algal blooms around the world, threatening drinking water supplies and public and environmental health. However, *Microcystis* genomes also harbor numerous biosynthetic gene clusters (BGCs) encoding the biosynthesis of other secondary metabolites, including many with toxic properties. Most of these BGCs are uncharacterized and currently lack links to biosynthesis products. However, recent field studies show that many of these BGCs are abundant and transcriptionally active in natural communities, suggesting potentially important yet unknown roles in bloom ecology and water quality. Here, we analyzed 21 xenic *Microcystis* cultures isolated from western Lake Erie to investigate the diversity of the biosynthetic potential of this genus. Through metabologenomic and *in silico* approaches, we show that these *Microcystis* strains contain variable BGCs, previously observed in natural populations, and encode distinct metabolomes across cultures. Additionally, we find that the majority of metabolites and gene clusters are uncharacterized, highlighting our limited understanding of the chemical repertoire of *Microcystis* spp. Due to the complex metabolomes observed in culture, which contain a wealth of diverse congeners as well as unknown metabolites, these results underscore the need to deeply explore and identify secondary metabolites produced by *Microcystis* beyond microcystins to assess their impacts on human and environmental health.

**IMPORTANCE:**

The genus *Microcystis* forms dense cyanobacterial harmful algal blooms (cyanoHABs) and can produce the toxin microcystin, which has been responsible for drinking water crises around the world. While microcystins are of great concern, *Microcystis* also produces an abundance of other secondary metabolites that may be of interest due to their potential for toxicity, ecological importance, or pharmaceutical applications. In this study, we combine genomic and metabolomic approaches to study the genes responsible for the biosynthesis of secondary metabolites as well as the chemical diversity of produced metabolites in *Microcystis* strains from the Western Lake Erie Culture Collection. This unique collection comprises *Microcystis* strains that were directly isolated from western Lake Erie, which experiences substantial cyanoHAB events annually and has had negative impacts on drinking water, tourism, and industry.

## INTRODUCTION

The cyanobacterium *Microcystis aeruginosa* threatens freshwater systems around the world (1, 2) as it regularly dominates cyanobacterial harmful algal blooms (cyanoHABs) and produces a wide range of secondary metabolites (Fig. S1)—with a range of toxic properties (3–5). CyanoHABs are expected to intensify with climate change through increased temperatures, rising atmospheric carbon dioxide concentrations, and exacerbation of anthropogenic nutrient pollution by extreme weather events (6–8). Some studies suggest that these conditions will favor more toxic cyanobacteria in future blooms (9–11). CyanoHABs pose threats to tourism, industry, access to potable water, and environmental and human health. Thus, it is critical to assess and monitor the threats of cyanoHABs under varying environmental conditions.

*Microcystis* spp. receive much attention for their production of the hepatotoxin microcystin (12, 13), which has led to several drinking water crises in both developed and developing nations (14–16), livestock poisoning and death (17), and human illness (18, 19). The vast majority of research on cyanobacterial secondary metabolism is devoted to the study of microcystins, which make up over 90% of the published literature (20). However, several novel cyanobacterial metabolite classes and their associated gene clusters have been discovered recently (21, 22), highlighting our limited understanding of their biosynthetic potential. Furthermore, *Microcystis* has complex genomes (23, 24) containing numerous biosynthetic gene clusters (BGCs) that may produce a diverse arsenal of secondary metabolites that are highly varied among species and strains (23–26). Many of these previously described secondary metabolites have toxic properties, which may have greater potency when produced in combination, and have been detected in drinking water sources (3, 20, 27–29). While some studies have characterized BGCs present in *Microcystis* strains (5, 25, 26) and others have resolved the structure and formula of metabolites including microcystins, aeruginosins, cyanopeptolins, and microviridins (30–35), studies that integrate genomic and metabolomic approaches to link genotype and chemical phenotype are lacking. As a result, our understanding of *Microcystis* secondary metabolism beyond microcystin and a few other cyanopeptides with toxic properties remains limited.

Western Lake Erie (WLE) is a critical source of freshwater for both the United States and Canada. CyanoHABs have become an increasing threat to WLE over the past 30 years as *Microcystis* dominates blooms on an annual basis (36–38). Current routine monitoring for four cyanotoxins in WLE includes microcystin, anatoxin-a, cylindrospermopsin, and saxitoxin (39). While these monitoring strategies are more extensive than in most freshwater systems, they may miss other potentially harmful secondary metabolites produced by *Microcystis* as evidenced by the abundance of transcriptionally active BGCs in natural WLE populations (26). Therefore, it is critical to deeply examine the secondary metabolite potential of *Microcystis* to define the extent of metabolite diversity and potential toxicity.

In this study, we leverage the Western Lake Erie Culture Collection (WLECC) (40) to better understand the repertoire of known and novel BGCs and secondary metabolites in various strains of *Microcystis* that comprise natural populations in WLE. Previously, the only cultivated and publicly available strain of *M. aeruginosa* isolated from WLE was LE-3 (41), which was isolated over 20 years ago. The WLECC is ideal for targeted WLE studies as it contains strains with various biosynthetic genotypes that were exclusively isolated from this location (40). Here, we deeply annotate BGCs encoding secondary metabolites, implement chemical profiling approaches to qualitatively assess chemical diversity produced in culture, and putatively link BGCs and metabolites via computational approaches to better understand the secondary metabolism of *Microcystis*.

## RESULTS

### Overview of WLE microcystis strain biosynthetic capacity

**Fig 1:**
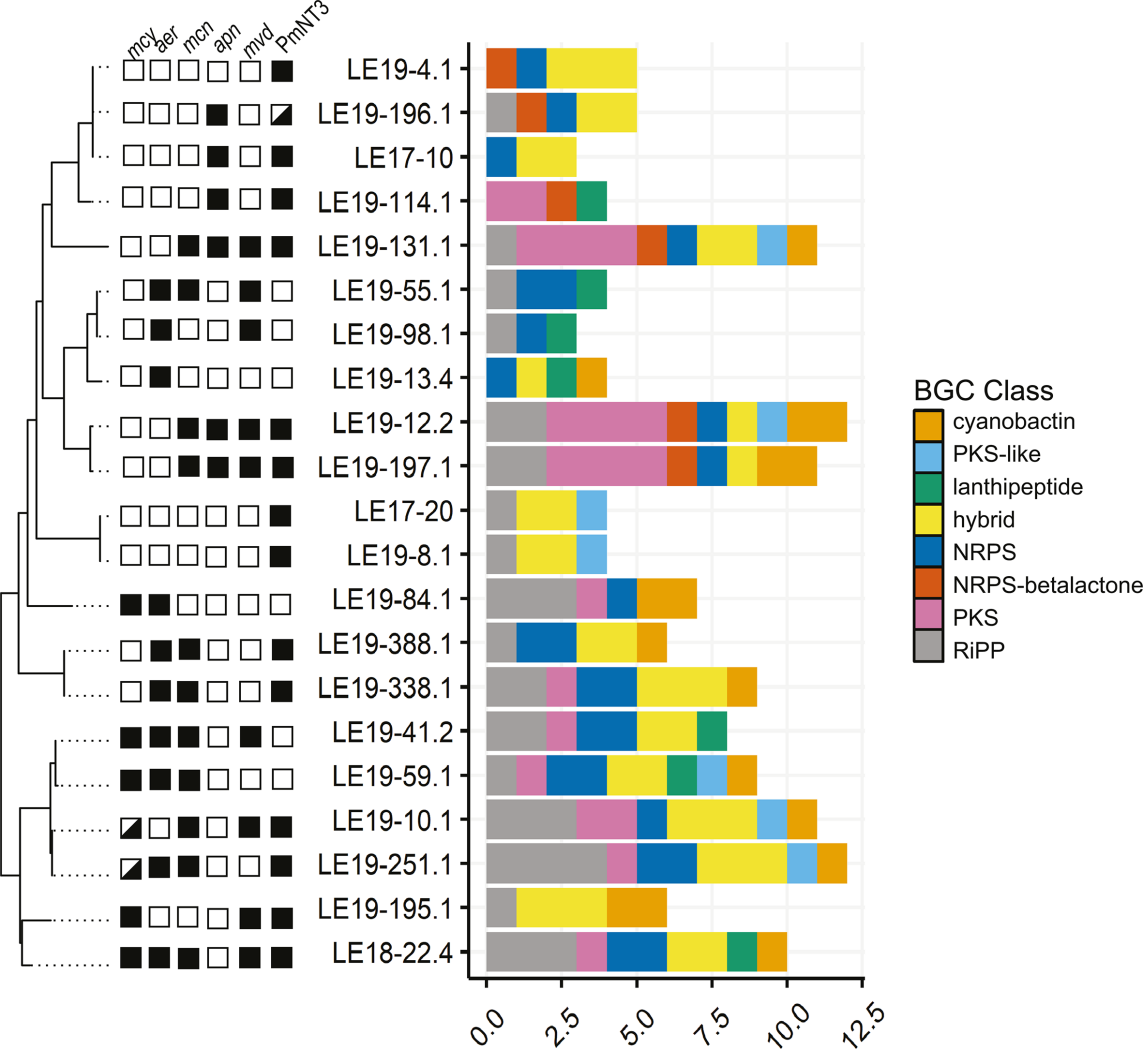
Overview of BGCs in WLE *Microcystis* culture isolates. A phylogenetic tree based on concatenated universal single-copy genes, of the *Microcystis* isolates in the WLECC, as previously described by Yancey et al. (43). Colored boxes indicate that the presence or absence of common *Microcystis* secondary metabolite genes includes the *mcy* (microcystin), *aer* (aeruginosin), *mcn* (cyanopeptolin/micropeptin), *apn* (anabaenopeptin), and *mvd* (microviridin B) operons, as well as the BGC, PmNT3, which may encode the biosynthesis of a paracyclophane molecule. The bar graph indicates the putative count and broad classification of BGCs within each *Microcystis* genome.

Genomic analysis showed that the WLE *Microcystis* strains contained diverse BGCs that encode multiple enzyme classes including nonribosomal peptides synthases (NRPSs), polyketide synthases (PKSs), hybrid NRPS-PKS modules, and ribosomally synthesized and post-translationally modified (RiPP) peptides. Of the 21 strains examined (Table S1), only five contained the complete *mcy* operon, which produces microcystin (Fig. 1), while two strains contained the partial *mcy* operon (42). Ten isolates contained complete BGCs thought to encode aeruginosins (*aer*), while 11 contained cyanopeptolin-encoding gene clusters (*mcn*). The *apn* operon, which encodes the biosynthesis of anabaenopeptins, was detected in six strains, while *mvd* genes encoding microviridins were identified in nine strains. These findings are consistent with previous results based on marker gene analysis (40). BGCs for microcystin, aeruginosin, or cyanopeptolin co-occurred in nine strains, while microviridin and an uncharacterized BGC containing two modular PKSs, an NRPS-like, and a T3PKS (PmNT3) encoding genes showed a more variable distribution among strains. Notably, strains with fewer NRPS or hybrid gene clusters were enriched in PKS and RiPP gene clusters. While BGC composition was relatively conserved within the deepest branches of the phylogenetic tree based on single-copy conserved genes (for example, LE17-20 and LE19-8.1), the count and type of BGCs varied within some clades (Fig. 1). Detailed gene annotations for each BGC are described in Table S2.

**Fig 2:**
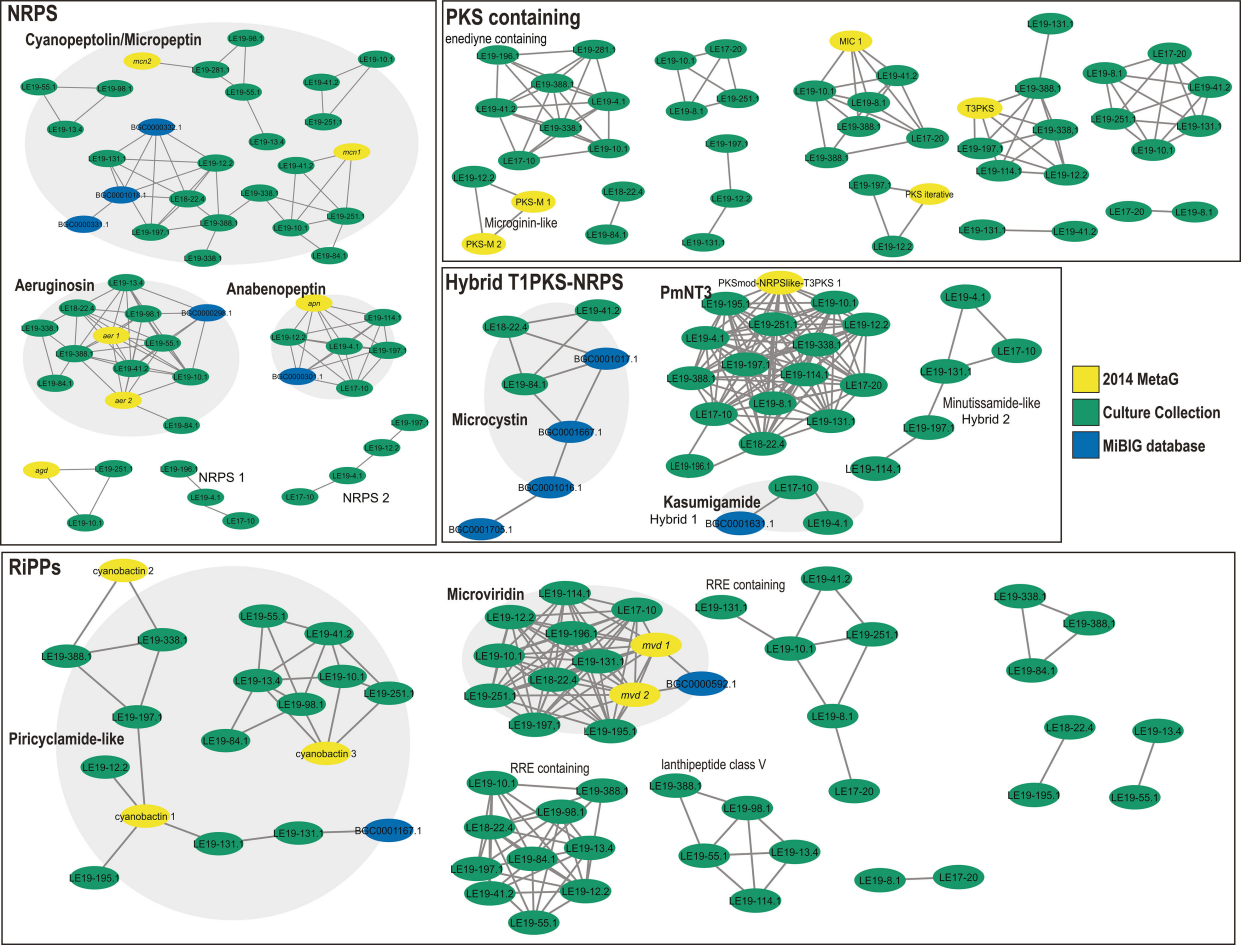
GCFs for Identified BGCs via BiG-SCAPE: Green nodes are from the WLECC, and yellow nodes are from the 2014 WLE cyanoHAB metagenomes. Nodes in purple are from the MiBIG database, which links BGCs with biosynthesis products. Edges between the nodes indicate the similarity of BGCs as calculated through a distance matrix that includes indexes that measure the number of shared PFAM domains, pairs of adjacent PFAM domains, and sequence similarities between predicted protein sequences.

We next investigated the relatedness of BGCs through the generation of gene cluster families (GCFs). We also included BGC sequences obtained from ten 2014 WLE cyanoHAB metagenomes (26) and sequences deposited onto the Minimum Information about a Biosynthetic Gene Cluster (MIBiG) (44), which contains BGCs with confirmed biosynthetic products. Clustering revealed that some GCFs contained BGCs known to encode the biosynthesis of cyanopeptolin/micropeptin, aeruginosin, anabaenopeptin, microcystin, and microviridin B (30, 31, 35, 45–47). GCF networks also illustrated that culture collection BGCs are representative of those observed in the field during the 2014 WLE cyanoHAB but also captured GCFs not identified in those 2014 metagenomes. The majority of GCFs do not contain MiBIG nodes, indicating the lack of previously described associated secondary metabolites (Fig. 2). Detailed GCF trees and cluster schematics for BGCs encoding known *Microcystis* secondary metabolites and uncharacterized PKS clusters are shown in Fig. S2 and S3.

### An unknown BGC with [7,7] paracyclophane biosynthesis potential

**Fig 3:**
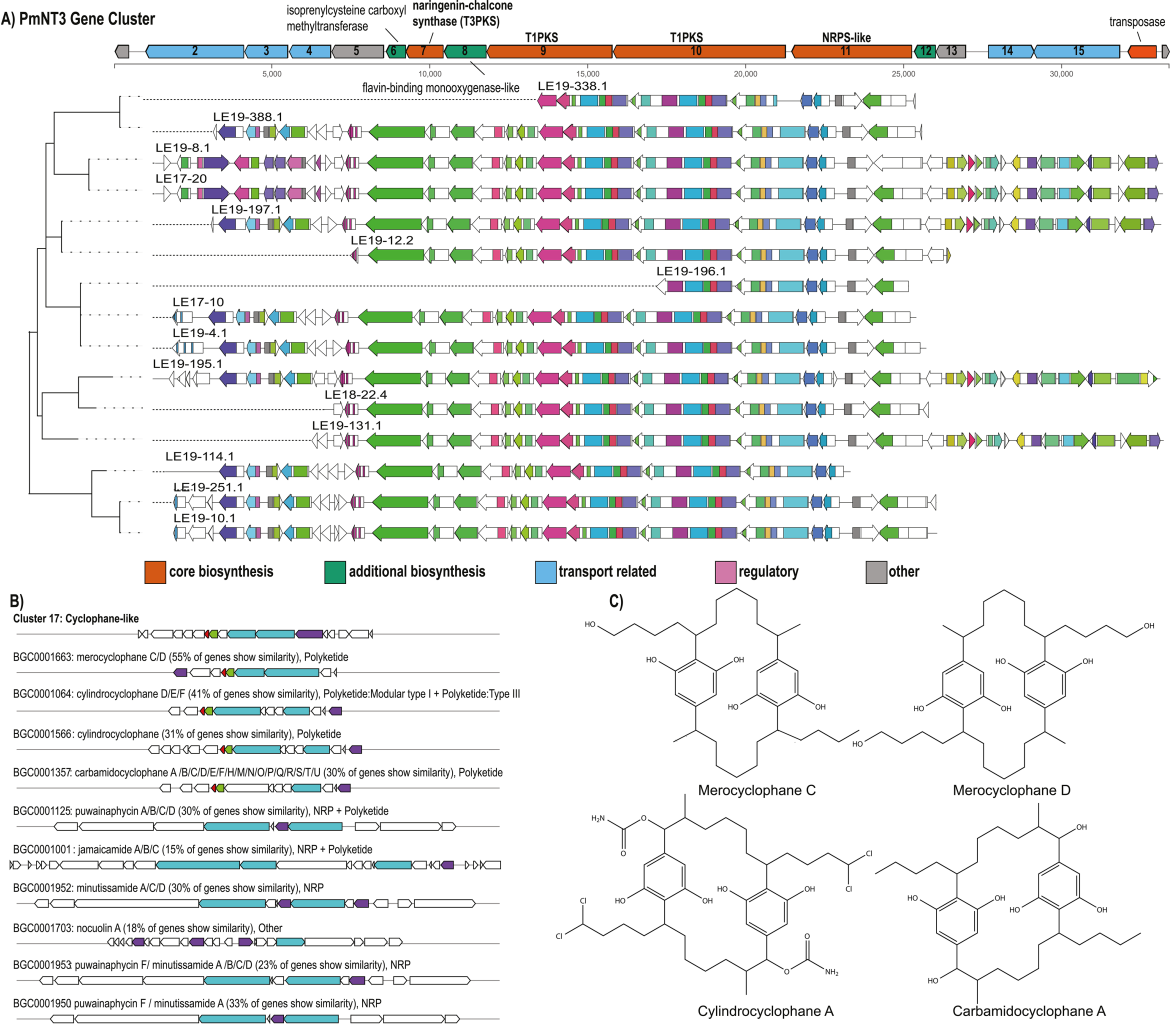
Gene schematics, gene annotations, and putative-related biosynthetic products of cryptic BGC *PmNT3*. (**A**) Detailed gene schematic and generated GCF tree from antiSMASH and BiG-SCAPE, respectively. Key genes are annotated on the large gene schematic. (**B**) Known cluster BLAST generated via antiSMASH. The novel BGC shows 30%–55% similarity of gene content to known cyanobacterial BGCs encoding for the biosynthesis of [7, 7] paracyclophanes: merocyclophane C/D (55%), cylindrocyclophane (41%), and carbamidocyclophane (31%). (**C**) Chemical structures for select cyanobacterial [7, 7] paracyclophanes that are confirmed biosynthesis products from the order *Nostacales*.

An unknown BGC, PmNT3, previously described as an “orphan” BGC in *M. aeruginosa* isolate PCC 7806 (23), was most closely related to BGCs that biosynthesize [7,7] paracyclophanes. This gene cluster, which encodes two modular PKSs, NRPS-like, and T3PKS enzymes, was present in 14 strains and partially present in one strain (LE19-196.1) (Fig. 1 and 3A). This cluster also contained biosynthesis genes that putatively encode an isoprenylcysteine carboxyl methyltransferase and a flavin-binding monooxygenase-like enzyme. Known cluster BLAST analysis (which identifies all genes to closest known BGC from the MiBIG database with significant BLAST hits) revealed 55% similarity to clusters that encode merocyclophane C/D (48), a 41% similarity to cylindrocyclophane D/E/F (49), and a 31% similarity to carbamidocyclophane (50) (Fig. 3B), all of which produce cyanobacterial [7,7] paracyclophane molecules (48, 49, 51, 52) (Fig. 3B and C). Genes with similarity hits between PmNT3 and known biosynthesis pathways for [7,7] paracyclophanes included core biosynthesis genes that encode both PKSs, the NRPS-like, T3PKS, and a methyltransferase as determined by antiSMASH (Fig. 3B). Despite its abundance in the WLECC and presence in the 2014 cyanoHAB (Fig. 2), only five publicly available *Microcystis* spp. strains contain this cluster at a 95% identity and query cover: NIES-298, PCC 7806, FACHB-905, PCC 7806SL, and FACHB-1751 (Genbank accessions: CP046058.1, CP020771.1, CP089094.1, AM778955.1, and CP011339.1).

A deeper examination and comparison of this putative gene cluster and the BGC that encodes the biosynthesis of merocyclophane C/D (48) revealed the presence of several conserved and core genes required for [7,7] paracyclophane biosynthesis. PmNT3 11, an NRPS-like encoding gene, shares similar domains and proposed functionality to *merI* and *merA*, which are believed to activate and load the PKS-derived chain. There is high gene sequence similarity (~50%) between the putatively identified T1PKSs in the PmNT3 pathway and T1PKS observed in the *mer* pathway. The T3PKS catalyzes the formation of resorcinol rings, a key step in paracyclophane biosynthesis that has been described for cylindrocylcophane biosynthesis (49). PmNT3 6 and *merF* appear to encode similarly functioning enzymes with carboxyl methyltransferase activity (Table 1). Finally, PmNT3 8 encodes a putative flavin-dependent monooxygenase-like enzyme.

**TABLE 1:** Comparison between merocyclophane encoding BGC (*mer*) from *Nostoc* sp. UIC 10110 (NCBI GenBank KY379971.1) and PmNT3

Merocyclophane (*mer*) gene homolog	Gene from WLECC	% identity	% alignment	Identified domains	Proposed function
*merI*	*PmNT3* 11	49	45 (first half)	Co-enzyme A ligase, AMP binding, Acyl-CoA dehydrogenase, and PP binding	Fatty acid co-ligase activation and loading of PKS chain (PP binding)
*merA*	*PmNT3* 11	31	8 (at the end)		
*merC*	*PmNT3* 10	48	97	Ketosynthase, acyltransferase, dehydratase, ketoreductase, and PP binding	Installs malonyl-CoA unit and performs full reduction of the ketone to methylene (no alpha methylation like in mer)
*merD*	*PmNT3* 9	46	98	Ketosynthase, acyltransferase, dehydratase, ketoreductase, PP binding, and thioesterase	Installation of second malonyl coA unit
*merE*	*PmNT3* 7	49	100	NA	Add third malonyl CoA, catalyzes resorcinol ring formation
*merF*	*PmNT3* 6	50	93	NA	Isoprenylcysteine carboxyl methyltransferase
NA	*PmNT3* 8	NA	NA	NA	Flavin-dependent monooxygenase-like formation of the final macrocycle

To explore whether this enzyme could be involved in ring closure of the final macrocycle via halogenation and C-C bond formation in a manner analogous to the cylindrocyclophanes (48, 53), a homology model of PmNT3 8 was compared to functionally characterized flavin-dependent halogenases (FDHs) and flavin monooxygenases (FMOs; Fig. S4). This analysis suggested that PmNT3 8 is most likely a monooxygenase (2.1 Å^2^ rmsd AncFMO5, PDB 6SEK). However, high structural homology (3.3 Å^2^ rmsd) with the single-component flavin-dependent halogenase AetF (PDB 8CJF) (54) was also observed. Halogenation of linear alkane substrates by FDH enzymes has not been previously reported, indicating that further studies are required to determine the catalytic function of PmNT3 8.

### Chemical profiling of the WLECC

**Fig 4:**
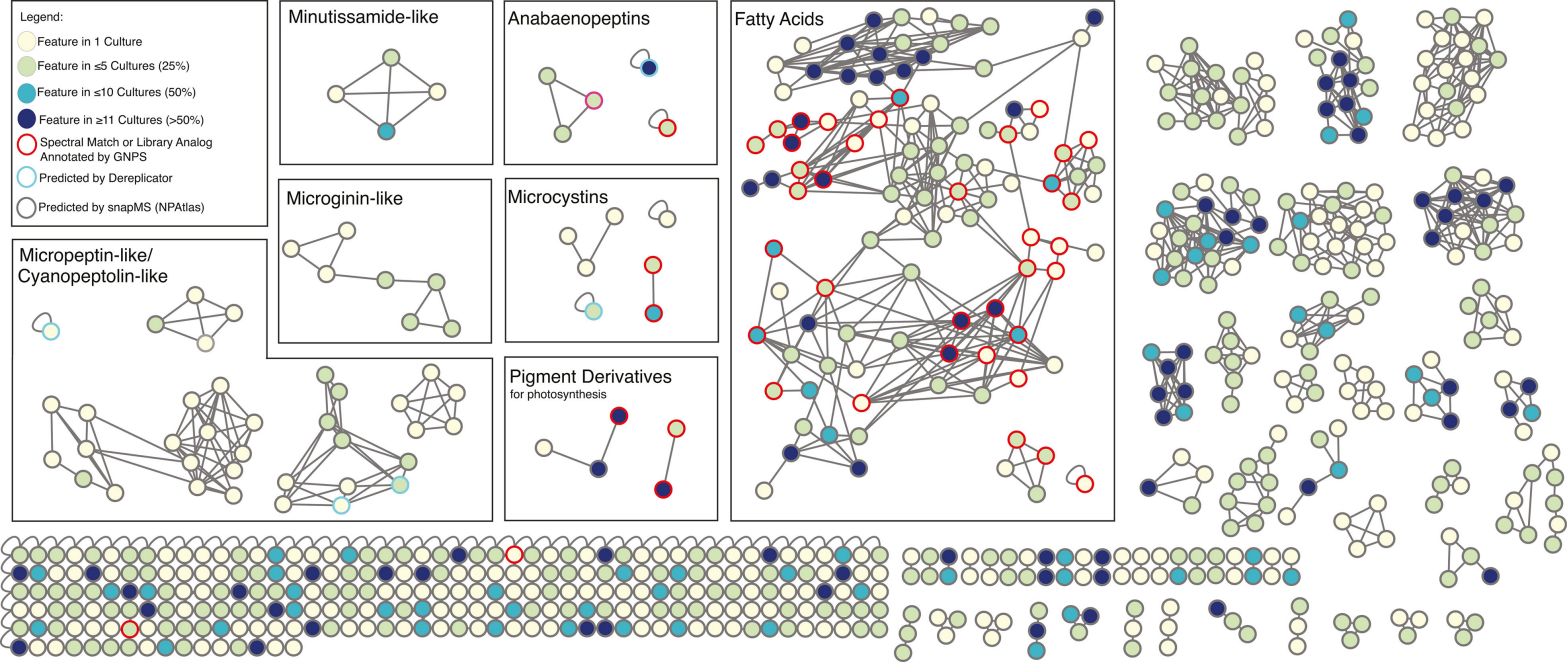
Molecular network representing the WLECC metabolome from positive mode liquid chromatography-mass spectrometry (LC-MS). Nodes are colored based on feature frequency within WLECC cultures (ranging from being found in one culture to all analyzed cultures). Metabolites annotated by GNPS, DEREPLICATOR, and SNAP-MS are outlined in red, light blue, and gray, respectively. Several known *Microcysti*s secondary metabolites were putatively identified including microcystins, anabaenopeptins, and features with similarity to micropeptins/cyanopeoptolins, microginins, and minutissamides.

Chemical profiling by mass spectrometry revealed a diverse metabolome among *Microcystis* isolates in the WLECC. Many chemical features are present in only one to five culture isolates (Fig. 4), highlighting highly variable metabolomes across cultures. Most chemical features do not have a spectral match or library analog to chemical structures deposited in the Global Natural Products Social Molecular Networking (GNPS) web server (55), but some putative identifications can be made. For example, several fatty acids, photosynthetic pigment derivatives, and microcystin congeners were annotated with GNPS. Mirror plots and manual annotations for microcystin LR, microcystin YR, and anabaenopeptin NZ857 to known spectral patterns and MS1 *m/z* values in the cyanometDB (56) provide confidence in the annotation of these cyanopeptides. Common fragmentation patterns are depicted in the manual annotations in all three compounds (Schymanski level 2, Fig. S5). Using additional bioinformatic annotation *in silico* tools DEREPLICATOR (57) and Structural similarity Network Annotation Platform for Mass Spectrometry (SNAP-MS [58]), we also putatively identified features for anabaenoepeptins, features with high similarity to microginins, and cyanopeptolins/micropeptins (30, 59, 60) (Fig. 4). These annotations are considered level 4 in the Schymanski labeling scheme, limiting our confidence in their actual structure and requiring additional data collection and manual annotation of these metabolites (61). Some chemical features, such as fatty acids and pigment derivatives, were detected often across strains (present in 11 or more cultures), while other features, such as microginin-like metabolites, were rarely detected (i.e., in less than five cultures). This, along with the presence of numerous unique or rare chemical features, highlights the distinct nature of metabolomes across cultures (Fig. 4). Even with the implementation of multiple annotators to facilitate the prediction of structural classes, many chemical features remain unannotated with uncharacterized structure. A complete list of putatively annotated chemical features using GNPS, DEREPLICATOR, and SNAP-MS by culture isolate is shown in Table S3. Of the 68 metabolites putatively annotated, 37 were annotated at level 5 confidence, 28 were at level 4, and 3 were at level 2.

**Fig 5:**
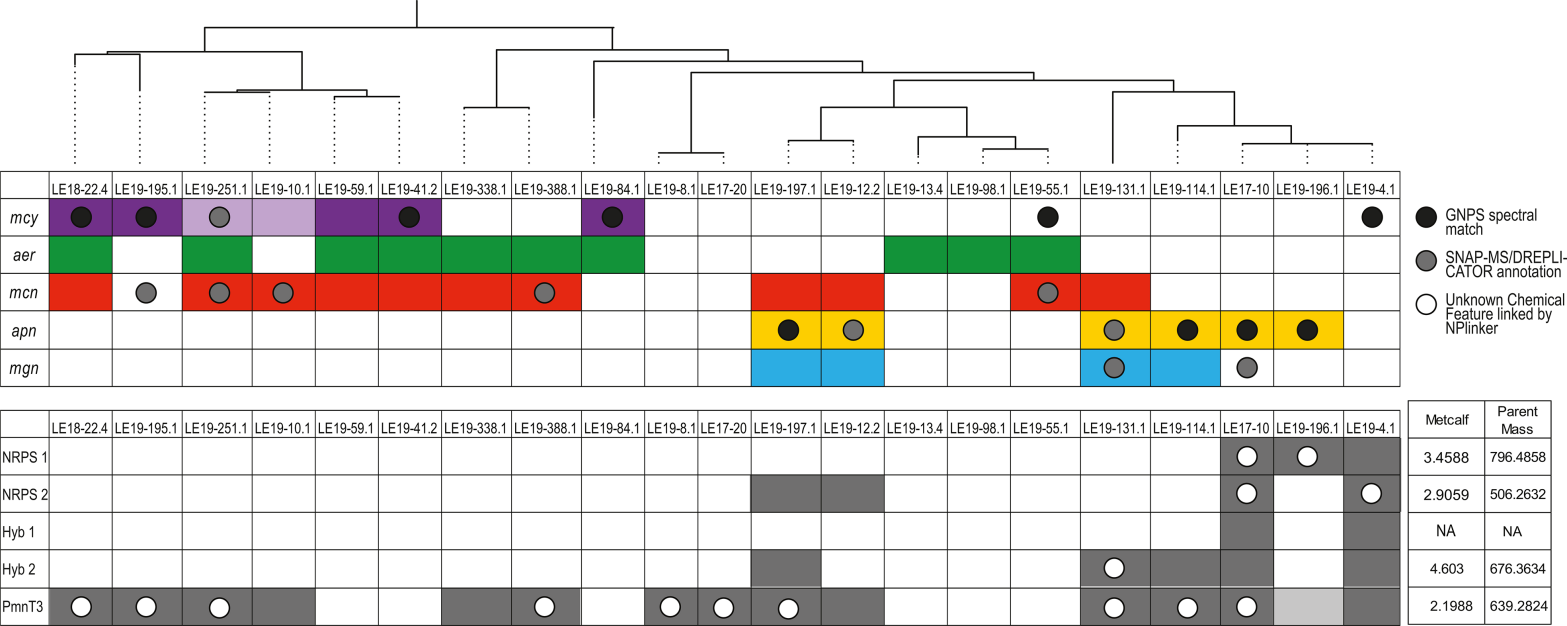
BGC and chemical feature absence matrix for select secondary metabolites: Cultures are arranged based on the phylogenetic tree generated via concatenated single-copy housekeeping genes as described in Yancey et al. (40, 43). On the top matrix, colored boxes indicate the presence of known BGCs (*mcy*, *aer*, *mcn*, *apn*, and *mgn* gene clusters). The light purple coloring indicates the presence of the partial *mcy* operon. Circles indicate the presence of an annotated chemical feature from each culture. Black circles were annotated with GNPS (highest confidence), and gray circles were annotated with either DREPLICATOR or SNAP-MS. On the bottom matrix, colored boxes indicate the presence of cryptic BGCs (annotated in Fig. 2). Gray coloring indicates the presence of core biosynthesis within the PmNT3 cluster in LE19-196.1 White circles indicate the presence of the top-scoring candidate chemical feature with respect to each BGC (NRPS 1, NRPS 2, Hyb1, Hyb 2, and PmNT3), as determined by NPlinker. The Metcalf score and parent mass for top hits are depicted in the box to the right.

We assessed the presence of a select set of BGCs and their corresponding annotated chemical features or top-scoring candidate features via NPlinker (62) in each culture (Fig. 5). In the case of the *mcy* and *apn* operons, which encode microcystin and anabaenopeptin, respectively, chemical features were annotated with GNPS in most cultures in which the BGC was present. The *aer* cluster was present in 10 cultures, yet there were no annotations for aeruginosin features in any culture (Fig. 5). Similarly, putative annotations for cyanopeptolins/micropeptins were observed in three out of the 11 cultures that contained the *mcn* gene cluster. This result supports the notion that BGCs are not constitutively expressed, and changing environmental conditions or available substrates may influence differential secondary metabolite biosynthesis (63).

For uncharacterized NRPS, hybrid, and PmNT3 clusters (Fig. 2), we also assessed the presence of top-scoring candidate features linked to respective BGCs via NPlinker. The chemical structures of these candidate molecular features are currently unknown. For both the NRPS 1 and 2 GCFs, two cultures with each respective BGC contained the top putatively linked chemical feature (Fig. 5). Of the five strains containing the Hybrid 2 GCF, only one strain contained the top putatively linked chemical feature. For the PmNT3 cluster, 10/14 strains with the complete BGC contained the top putatively linked chemical feature (Fig. 2 and 5). In some cases, microcystins, putatively identified cyanopeptolins/micropeptins, and microginins were annotated in cultures in which the respective BGC was not present (Fig. 5). This may be explained by observed *Microcystis* strain heterogeneity within a few WLECC cultures, in which relative abundance of strains may fluctuate over time during continuous cultivation (40).

### Diversity within cyanopeptolin/micropeptin BGCs and congener identified

**Fig 6:**
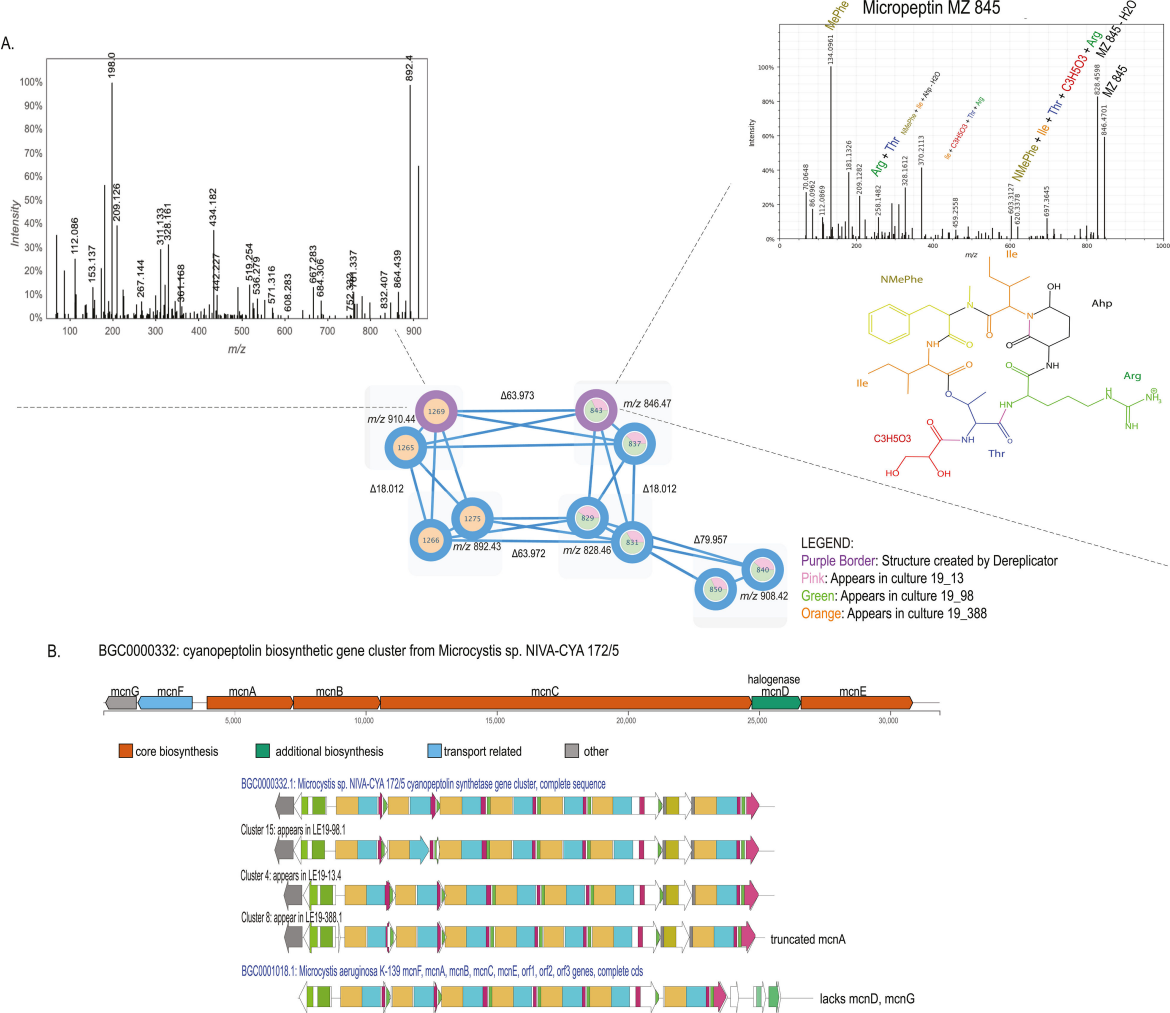
Cyanopeptolin/micropeptin molecular networking and gene cluster analysis. (A) The molecular network with micropeptin cluster was obtained from the WLECC with a cosine similarity score cutoff of 0.70. Nodes are labeled with corresponding cluster index numbers assigned through GNPS, and their *m/z* are shown in black text next to each pair of nodes that share the same *m/z*. Edges between nodes show the mass difference between sets of nodes. Two micropeptins with edges outlined in purple indicate GNPS probable structure detection through GNPS DEREPLICATOR. Pie charts within nodes indicate strains that the metabolites were detected in. Cluster index 843 corresponds to CyanoMetDB micropeptin MZ 845 shown in the figure with the experimental MS/MS [M + H]^+^ annotated data shown. Ahp is 3-amino-6-hydroxy-2-piperidone. Cluster index 1,269 had a predicted structure from GNPS DEREPLICATOR but does not correspond to experimental MS/MS data, and the structure remains unidentified. (**B**) Gene schematics for three gene clusters identified in three WLECC strains, and their comparison to known *mcn* gene clusters deposited in the Minimum Information about a Biosynthetic Gene Cluster (MiBIG) database (https://mibig.secondarymetabolites.org/repository). Genes are colored based on PFAM domain annotation for each cluster.

Three unique cyanopeptolin/micropeptin-like BGCs were identified in the isolates from this culture collection based on deduplication of sequences (see Materials and Methods, Fig. 6) These clusters were very similar to the *mcn* BGC from isolate *Microcystis* sp. NIVA-CYA (NCBI Genbank: DQ075244.1). Clusters 15 and 4 shared over 97% identity with *mcn* gene cluster from the NIVA-CYA strain, while Cluster 8 was 87% similar. All three WLECC clusters contain a halogenase (*mcnD*), unlike the *mcn* BGC from *M. aeruginosa* K-139 (NCBI Genbank: AB481215.1, Fig. 6B). Putative substituents for features representing micropeptins, specifically features 1,269 and 843, were identified using DEREPLICATOR. Feature 843 shared the *m/z* ratio of 846.47 with micropeptin MZ845, queuing subsequent manual annotation of these spectra. Manual annotation of these spectra verified the identity of this feature with level 2 confidence. Feature 1,269 has an MS1 pattern matching micropeptins HU909 and SF909 in CyanoMetDB (56), but isotope signatures verified no chlorine present in the structure, and therefore, the metabolite could not be further annotated with the available data (Fig. 6A). Four nodes are strictly found in LE19-388.1, and six nodes are found in both LE19_98.1 and LE19-13.4, highlighting chemical diversity of the metabolite class between strains.

### Putatively linking BGCs to spectra

**Fig 7:**
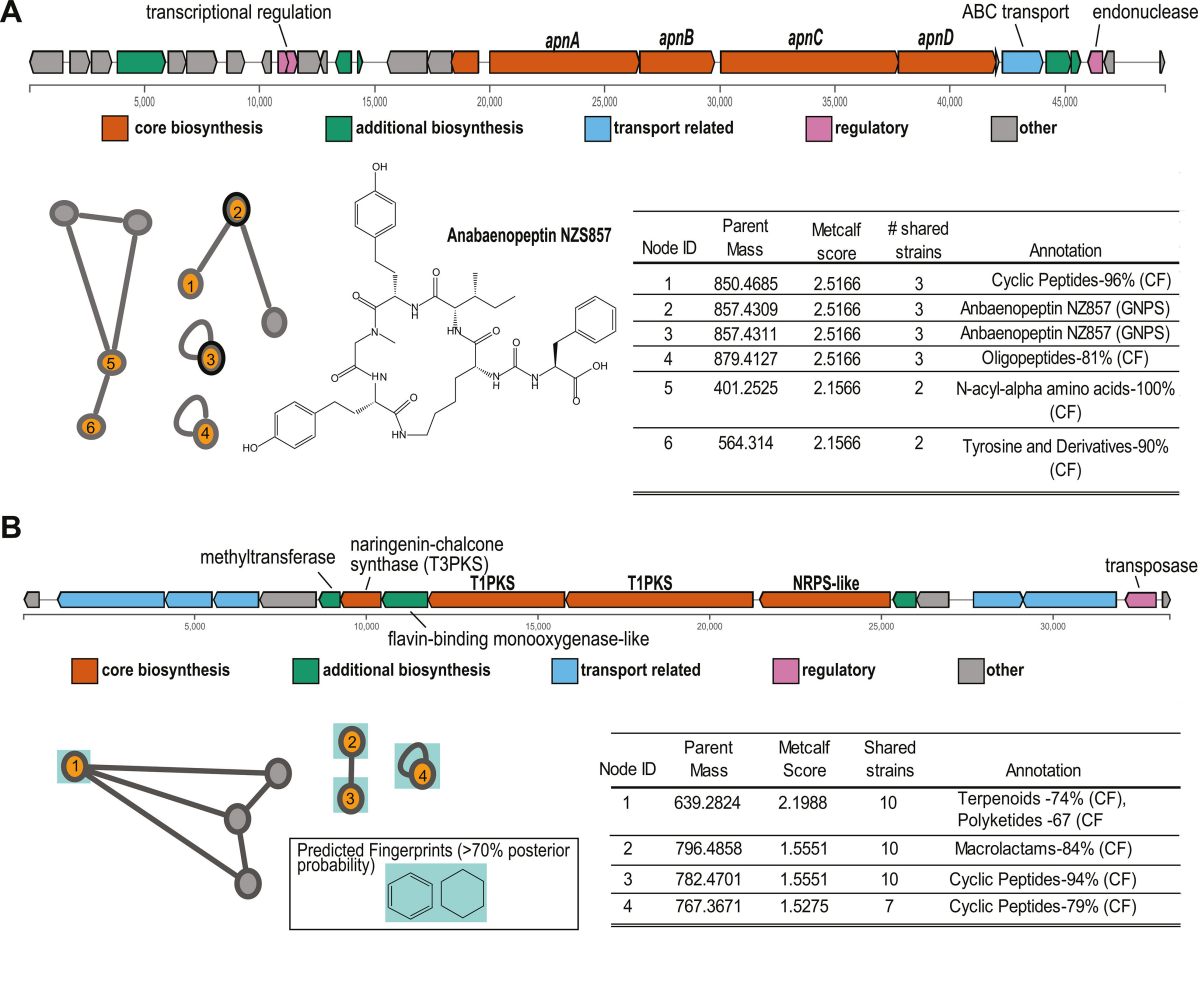
Spectra linked to the anabaenopeptin (A) *apn* and (B) PmNT3 gene clusters through NPLinker. Linked spectral features identified by NPlinker are colored orange, and the parent mass, Metcalf score, number of shared strains, and putative annotation via GNPS and/or ClassyFire are reported in the table. Gray nodes represent features that were not linked with NPlinker but were within subnetworks for putatively linked features. For the PmNT3 cluster, select molecular fingerprints including ring systems, halogenations, or sulfurous qualities are highlighted with differing colors.

To link candidate products and GCFs observed in the WLECC and putatively identify relationships between uncharacterized spectra and BGCs, *in silico* scoring approaches were used. For proof of concept, we assessed links between spectral data and the gene clusters putatively encoding anabaenopeptin (*apn*) and the PmNT3 cluster. NPlinker putatively linked six spectra to the *apn* gene cluster, including two annotated as anabaenopeptin NZ857 from the GNPS library database. In total, three features were identified as anabaenopeptins (two through GNPS, one through Sirius/ClassyFire), while an additional two features were coarsely identified as “Cyclic Peptides” using the Sirius workflow (Fig. 7A). These spectra may represent previously undescribed congeners of anabaenopeptin or structurally similar molecules outside of the anabaenopeptin class. Additionally linked candidate spectra received various generic annotations from Sirius/ClassyFire with variable confidence and may represent derivatives or intermediate synthesis products of anabaenopeptin, or false positives (Fig. 7A; Table S4). Detailed annotations, select molecular fingerprints, and structure predictions for putatively linked spectra are shown in Table S4.

For the cryptic PmNT3 GCF, NPlinker identified four features that potentially represent biosynthesis products. Based on the highest Metcalf score, the best match was annotated as a molecule with terpenoid/polyketide-like qualities (74%/67% confidence) using Sirius/ClassyFire (64, 65). Additionally linked candidate spectra were also annotated generically with variable confidence (Fig. 7B). While it is unlikely that these chemical features are 100% matches to predicted annotations, their structural properties share some similarities with intermediate and completely biosynthesized [7,7] paracyclophane-like molecules. Molecular fingerprint annotations provide evidence that putatively linked spectra to the PmNT3 gene cluster consist of at least one ring system, a key feature in paracyclophanes (48, 49, 52). Detailed annotations, select molecular fingerprints, and structure predictions for putatively linked spectra are shown in Table S5. While we are unable to resolve the complete structure of the biosynthesis product from PmNT3, and putatively linked spectra may represent false positives, these results suggest that biosynthesis products from this GCF may contain chemical properties of paracyclophane molecules (66). Complete NPlinker results, which putatively link all identified GCFs and associated chemical features, can be found at https://github.com/ceyancey/Metabalo-genomics-of-WLECC.

## DISCUSSION

We investigated *Microcystis* secondary metabolism through integrated analysis of BGCs and MS features of metabolites in cultures isolated from WLE. The cultures used in this study are not axenic, as they contain several bacteria known to associate with *Microcystis* in natural cyanoHAB communities (40, 67). Regardless, these cultures are simplified compared to environmental samples and thus valuable for laboratory study. While most WLECC cultures were isolated in 2017–2019 during early-to-peak phases of the bloom (40), they captured the genetic diversity of putative BGCs observed in the 2014 WLE cyanoHAB, as well as clusters not observed during that time (Fig. 1 and 2). By pairing metabolomics with genomic analyses, we identified known and novel secondary metabolites and putatively linked GCFs with chemical features through *in silico* approaches. Our study highlights several known BGCs, predicted biosynthesis products, and several uncharacterized clusters and features, revealing the vast breadth and diversity observed in *Microcystis* gene clusters and metabolomes that have yet to be described.

Both genomic and metabolomic analyses reveal the potential for several *Microcystis* strains to produce secondary metabolites with toxic properties, including microcystins, aeruginosins, cyanopeptolin/micropeptins, anabaenopeptins, and microginins (Fig. 1, 2, 4, and 6) (31, 33, 45, 68, 69). These results are consistent with the co-occurrence of secondary metabolites from *Microcystis*-dominated cyanoHABs in lakes and raw drinking waters (27, 28, 70). Growing evidence indicates that such combinations of multiple secondary metabolites can have compounding effects regarding bioactivity and/or toxicity (29). Our culture collection of *Microcystis* strains that contain variable suites of cyanopeptides within each culture (Fig. 4 and 5; Table S3) will be valuable in studying potential toxic and bioactive synergisms in mixtures of secondary metabolites. However, our results also emphasize that culture metabolomes provide a snapshot in time, and BGC presence is not indicative of a biosynthesis product, as different clusters may be expressed under different conditions (Fig. 5). Our untargeted approach also revealed greater congener diversity of various metabolites compared to approaches that employed standards for detection (27, 28, 70), although confidence in congener identity is lower without the use of standards. The greatest chemical diversity was observed for putatively identified cyanopeptolin/micropeptin features that were largely predicted by SNAP-MS (Fig. 4 and 6; Table S2). Level 3 confidence is reported for one of these micropeptin analogs due to further manual annotation (*m/z* 846.47), but we were not able to solve the structure of other micropeptins without further data collection or use of standards (61). Cyanopeptolins, which are found globally (20), inhibit serine proteases and chymotrypsin, which can have neurotoxic effects (3, 71, 72) and disrupt food webs (73, 74).

In addition to identifying biosynthesis pathways and chemical features from known *Microcystis* secondary metabolites, our approach enabled the exploration of novel BGCs, including those putatively encoding biosynthesis of a [7,7] paracyclophane-like molecule. These metabolites contain structural symmetry and insertion of various functional groups and are believed to derive from decanoic acid (48, 49, 52, 75). To date, [7,7] paracyclophanes have only been isolated from fungal (76) or cyanobacterial sources, but a biosynthesis product has yet to be identified from *Microcystis*. Previous work has identified the presence of the PmNT3 cluster in several *Microcystis* genomes (75). This BGC also has an inverse relationship in transcriptional activity with the *mcy* operon (77), suggesting that produced metabolites may have tradeoffs or serve similar functions under different environmental conditions. Based on the reported cytotoxicity of other [7,7] paracyclophanes (52) and high transcriptional activity in the 2014 western Lake Erie cyanoHAB (26), its biosynthetic routes, chemical structure, and functional role are a high priority for continued research.

The *Microcystis* PmNT3 cluster lacks homologs to *cylC* and *cylK* (78), which are essential for the formation of the final macrocycle, suggesting that a linear analog may be biosynthesized (53, 75). However, our results also show the presence of a putative flavin-dependent monooxygenase-like gene within the BGC (PmNT3 8; Fig. 3; Table 1) that may catalyze macrocycle formation through C-H functionalization (53). Additionally, *cylC* homologs have previously been identified in *Microcystis* genomes (53), and we identified a putative *cylC* homolog in 9 out of 15 isolates that contain the putative cyclophane-encoding BGC (Table S6), though they were not co-localized with the PmNT3 cluster. Homologs for *cylK* were not detected in any of the *Microcystis* genomes. Missing *cylC* and *cylK* homologs likely reflects true gene absence as paired-end read mapping failed to detect these genes (data not shown). Together, the presence of a putative flavin-dependent monooxygenase-like gene within the PmNT3 cluster, as well as the presence *cylC* homologs within *Microcystis* isolates, suggests the possibility of a novel mode of [7,7] paracyclophane biosynthesis, but more research is needed to ascertain chemical structure and the complete biosynthetic pathway.

*In silico* approaches yielded a concise list of candidate spectra linked to GCFs of interest. By linking the *apn* GCF and chemical features annotated as anabaenopeptins, we provide proof-of-concept for the utility of *in silico* linking between genomic and metabolomic data (Fig. 7A). We also made conservative inferences regarding the uncharacterized gene cluster PmNT3 and unknown metabolites. While these inferences provide valuable hypotheses, given the low confidence in structure prediction by Sirius (Fig. 7B; Table S5) and the potential for false positives from NPlinker (62, 79), these hypotheses should be tested with further studies. For example, chemical fingerprints provide evidence that putative products of the PmNT3 cluster contain characteristic functional groups of paracyclophanes such as ring systems, but overall structure predictions do not provide high confidence in the detection of a paracyclophane molecule (Fig. 7B). Genetic annotation of the PmNT3 cluster provides evidence that a synthesis product would have greater polyketide character than peptidic character (Fig. 3; Table 1), while some lower scoring, putatively linked spectra are peptidic in nature (Fig. 7B). Because of this, some links may represent false positives and highlight the need for traditional structural elucidation approaches. While applications such as nuclear magnetic resonance (NMR) and heterologous expression to determine exact biosynthetic mechanisms and produced products are beyond the scope of this study, the PmNT3 case study highlights the need for future molecular approaches to determine the structure of the final-produced metabolite. The WLECC is an excellent resource for elucidating the chemical structure of secondary metabolites such as those encoded in the PmNT3 cluster, which was heretofore considered “orphan” (23, 26, 75).

Our results demonstrate the diversity and wealth of uncharacterized *Microcystis* biosynthesis pathways and metabolomes. While this study highlights a few BGCs, substantial biosynthetic potential can be explored through the WLECC, as evidenced by the abundance of unknown and coarsely annotated gene clusters and chemical features. Each culture appears to have a distinct metabolome (Fig. 4 and 5; Table S2) despite the similar BGC compositions observed between closely related or identical strains (Fig. 1). Identifying drivers of differential metabolome compositions is beyond the scope of this study but may be a result of different associated bacterial assemblages within cultures and microbial exchange of various substrates (40, 80, 81), or subtle differences in genomic sequences, which may encode slightly variable enzyme complexes leading to expanded structural diversity (82). Ultimately, these results may be used to better understand observed cyanoHAB dynamics in WLE and inform monitoring and management strategies to mitigate the impacts of toxic secondary metabolite production.

## MATERIALS AND METHODS

### Western Lake Erie culture collection and sequencing

Collection, isolation, cultivation, and DNA extraction of *Microcystis* WLECC isolates have been recently described (40). Briefly, the WLECC consists of 21 xenic *Microcystis* isolates. Cultures were maintained in BG-112N medium, at 22.9°C, with a 12/12 hour light/dark cycle with light of 40 µmol photon/m^2^/s. DNA was extracted approximately 7 days after initial passaging with the DNeasy Blood and Tissue Kit (Qiagen, Hilden, Germany) and QIAShredder adapter (Qiagen, Hilden Germany) according to this protocol: https://www.protocols.io/view/dna-extraction-from-filters-using-qiagen-dneasy-an-jx5cpq6. DNA concentration was determined using the QuantIT PicroGreen dsDNA Assay Kit (Fisher Scientific, Waltham, MA, USA). Extracted DNA was sequenced at the University of Michigan Advanced Genomics Core (Ann Arbor, MI, USA) using the NovaSeq S4 platform (Illumina, San Diego, CA, USA) with 300 cycles. Paired end sequences were run with the maximum library fragment (insert) size possible without compromising read quality.

### Bioinformatic analysis

Raw sequence reads were quality checked and processed using bbduk, from the Joint Genome Institute (JGI)-supported suite of tools, bbtools (83), to remove adapters, trim reads for quality, and remove contamination using the UniVec reference collection (https://www.ncbi.nlm.nih.gov/tools/vecscreen/univec/). Duplicate reads were removed using dedupe as part of the bbtools version 38.84 package as well. Individual *de novo* assemblies were completed for each isolate using Megahit v.1.2.9 with the meta-sensitive parameter (84). Contiguous sequences of at least 1,000 base pairs in length were kept for downstream analysis including creating an Anvi’o v.7 (85) database for each isolate. Each database was manually refined into *Microcystis* and associated bacteria metagenome-assembled genomes (MAGs) for each culture (40).

Each *Microcystis* MAG was then run through AntiSMASH v.6.0.1 (86) with default parameters under relaxed settings. BGCs were further analyzed if they were at least 5.5 kb in length, and contained NRPS, PKS, multi (NRPS and PKS combinations), microviridin, cyanobactin, lanthipeptide, RiPP, or spliceotide annotation via antiSMASH. BGCs annotated as terpenes were excluded from further analysis. A deduplicated list of BGCs was subjected to deep annotation, including gene-by-gene annotation using PFAM, TIGRfam, and blastP (Table S1). GCF analysis was completed using BiG-SCAPE (87) with default parameters.

To determine which *Microcystis* genomes contained which BGCs and their degree of completion, a series of mapping approaches were used. This was done due to the quality of *Microcystis* MAGs, which are considered drafts genomes, with variable N50s due to low coverage obtained through sequencing (40). First, BGC sequences were aligned to genomes using BLAST (88). Positive hits were considered if the following criteria were met: at least 95% identity, at least 80% alignment length, and e-value no greater than 1 × 10^−5^. For further confirmation of BGC presence in a particular *Microcystis* genome, especially for ambiguous alignments that did not satisfy the above parameters, read mapping and visualization were also completed. Briefly, bbmap from the JGI-supported bbmap tools package (83) was used to map QC reads from each *Microcystis* isolate onto BGCs setting the minID to 0.95, the IDfilter to 0.8, and allowing ambiguous reads to multimap. Read mapping alignments were then visually inspected in Tablet (89) to determine the presence or absence of core biosynthetic genes for each cluster based on the evenness of coverage patterns (see Fig. S5 for an example). This ensured the detection of complete BGCs that assembled poorly such as the *mcy* and *mcn* operons in some *Microcystis* isolates.

### Protein modeling

A homology model for PmNT3 8 was generated using SWISS-MODEL (90) software with the AlphaFold structure AF-A0A552JBG4-F1 (99.3% sequence identity with PmNT3 8) as a starting model. The PmNT3 8 homology model was used to query the protein data bank for structural homologs using the DALI server (91). Superposition of the PmNT3 8 homology model with AncFMO5 and AetF was performed using LSQ superpose in Coot (92). The figures were generated using PyMOL (Schrödinger).

### Mass spectrometry sample preparation and data acquisition

The following steps were completed for mass spectrometry sample preparation and data acquisition. All 21 cultures were grown in 50 mL of BG11-2N medium for 2 weeks. Once sufficient biomass was achieved, cultures were filtered onto 47 mm GF/F filters (Whatman, Maidstone, UK). Filters were stored at −80°C until further analysis. Extractions were performed using a 50:50 HPLC grade methanol/chloroform extraction protocol. Briefly, thawed GF/F filters were suspended in 50:50 methanol/chloroform solution and sonicated for 30 minutes. The extraction was then mixed gently for 4 hours at 30°C, filtered to remove solids, dried under nitrogen, and stored at −20°C until further analysis.

Ultra-high-performance liquid chromatograms (UHPLC) were obtained on an Agilent liquid chromatography-mass spectrometry system made up of an Agilent 1290 Infinity II UHPLC coupled to an Agilent 6545 ESI-Q-TOF-MS in positive ionization mode. Aliquots (1 µL) of the WLECC *Microcystis* extracts (1 mg/mL in methanol) were analyzed on a Kinetex phenyl-hexyl (1.7 µm, 2.1 × 50 mm) column using the described parameters and method in references (93). Briefly, a 1-minute isocratic elution of 90% A (A = 100% H_2_O and 0.1% formic acid) followed by 6-minute linear gradient elution to 100% B (B = 95% acetonitrile, 5% H_2_O, and 0.1% formic acid) with a flow rate of 0.4 mL/min was used followed by isocratic elution with 100% B for 2 minutes. The electrospray ionization (ESI) system was calibrated such that the capillary temperature was set to 320°C, the source voltage was 3.5 kV, and the gas flow rates were 11 L/min. Ions above intensity 1,000 were detected and counted at 6 scans/s with a maximum of nine selected precursors per cycle using ramped collision energy (93). Data-dependent analysis was performed. The acquired MS/MS data were converted from Agilent (Agilent, Santa Clara, CA, USA) vendor data files (.d) to mzXML file format using MSConvert software through the Proteowizard package (94).

### Analysis and annotation of MS/MS data

A suite of *in silico* tools was used to process and analyze the mass spectrometry data to identify and annotate “features,” which are defined as an ion signal of a specific mass-to-charge ratio (*m/z*) detected at a specific time (retention time-RT) that has an accompanying MS/MS spectrum available (95). First, MZMine v2.53 (96) was utilized for feature extraction and alignment across all WLECC extract MS samples, with alignment based on unique features across different samples being linked to a specific RT and *m/z*. In MZMine v2.53, initial mass detection and filtering parameters were set with the MS1 threshold being 1e03 and the MS2 threshold being 1e02. Chromatogram building utilized a minimum group size number of scans as 2, a group intensity threshold of 2e04, a minimum highest intensity of 5e03, and *m/z* tolerance of 10 ppm. Chromatogram deconvolution utilized a chromatographic threshold of 50%, a search minimum RT range of 0.20 minutes, a minimum relative height of 10%, a minimum absolute height of 5e0, and a peak duration range 0–3 minutes, an *m/z* range for MS2 scan pairing of 0.1 Da, an RT range for MS2 scan pairing of 0.2 minutes, and a local minimum search algorithm. For isotope grouping, a *m/z* tolerance of 10 minutes, RT tolerance of 0.05 minutes, and a maximum charge of 2 were used. Alignment was conducted with a weight of 75 for *m/z* ratio and 25 for RT and an RT tolerance of 0.05. Downstream filtering in MZMine took place and is described below. In total, 2,784 features were extracted from positive and negative ionization mode MS/MS data and annotated with unique identification numbers. Associated feature intensities in each sample were identified based on peak areas in extracted-ion chromatograms. Features corresponding to extraction solvent and uninoculated growth medium were removed from the feature set, resulting in 1,307 total features in positive ionization mode for analysis.

Feature-based molecular networking (FBMN) through the GNPS (55, 95) was used to identify subnetworks of structurally related features. Connections (edges) and features (nodes) within the subnetworks can be visualized with Cytoscape software (97). Additionally, GNPS identifies spectral matches (exact or analogous metabolites) in submitted MS/MS data by searching through publicly available databases of MS/MS data, which were annotated in the Cytoscape molecular network. DEREPLICATOR, built into the GNPS platform, was used for additional annotation of the molecular network in order to predict peptidic secondary metabolites with a *P*-value 5 × 10^−12^ or lower (57). Results for GNPS analysis (both positive and negative ionization mode) can be found at https://gnps.ucsd.edu/ProteoSAFe/status.jsp?task=64896147136e41ffaf17176f3d90937e and https://gnps.ucsd.edu/ProteoSAFe/status.jsp?task=36964d2641364f658e30f32629e85081, respectively. Lastly, annotations from SNAP-MS were used to assign compound families to subnetworks in the molecular network using groupings in the Natural Products Atlas (58, 98, 99). Final data outputs were visualized in Cytoscape v 3.9.1 (97). Annotations with spectral matches to databases in GNPS were given a level 3 annotation, and those annotations which had manual inspection and verification from this set were labeled as level 2 confidence, according to Schymanski principles (61). Annotations made by DEREPLICATOR and SNAP-MS were annotated as level 5. Manual annotations of the cyanopeptides were conducted using library reference mirror plots from GNPS (Fig. S5) and manual annotations of mass changes corresponding to amino acids and other fragments of the molecule (Micropeptin MZ 845). CyanometDB was utilized to verify the MS1 and molecular formula of the manually annotated metabolites (56).

### *In silico* putative linking of GCFs and spectral data

The software NPlinker was used to putatively link BGCs with spectral data (62). NPlinker requires outputs from BIG-Scape, AntiSMASH, and GNPS to putatively link candidate synthesis products and GCFs. The NPlinker analysis was run on Docker according to the workflow described at https://github.com/NPLinker/nplinker-webapp. We report putative links between GCFs and spectral data, along with the parent mass, number of shared strains, and Metcalf scores for *apn* and PmNT3 clusters. Putatively linked spectra to GCFs of interest were additionally annotated using the SIRIUS workflow (65, 100, 101).

### Statistical analysis and figure rendering

Figures were generated in R on R Studio (102) using the packages ggplot2 (103) and ggpubr (104). GCF networks and BGC gene schematics were generated in BiG-SCAPE (87) and edited further for visual clarity in Cytoscape (97). Metabolomic networks were generated by GNPS and further refined in Cytoscape as well.

## Data Availability

Raw reads and metagenome assembled genomes (MAGs) were deposited at the respective NCBI repositories and are available in BioProject PRJNA903891. Raw reads are available under the SRA accession numbers: SRR22360640–SRR22360660. Raw mxXML files for metabolomic data can be found at https://massive.ucsd.edu/ProteoSAFe/dataset.jsp?task=cd03648b2a174e4697c123db59db40bf.

## SUPPLEMENTAL MATERIAL

Supplemental Figures msystems.00334-24-s0001.docx Supplemental table captions and Figures S1 to S6.

Supplemental Tables msystems.00334-24-s0002.xlsx Tables 1–6.

ASM does not own the copyrights to Supplemental Material that may be linked to, or accessed through, an article. The authors have granted ASM a non-exclusive, world-wide license to publish the Supplemental Material files. Please contact the corresponding author directly for reuse.
