# Supplementary material for: Metabologenomics reveals strain-level genetic and chemical diversity of Microcystis secondary metabolism
Source: mSystems. 2024 Jun 25;9(7):e00334-24. doi: 10.1128/msystems.00334-24 (PMC11264947; doi:10.1128/msystems.00334-24)
Supplement: Supplemental Figures — Supplemental table captions and Figures S1 to S6. [file msystems.00334-24-s0001.docx]

**Supplementals**

All tables can be found in the excel sheet.

Table S1: Summary of WLECC strains used for this study. Isolation method, media, and year are listed in addition to the dominant mcy genotype found in each culture. For more information please see reference 40.

Table S2 – Gene by gene annotations for dereplicated BGCs identified in the WLECC

Table S3: Chemical feature annotations from GNPS, DEREPLICATOR, and SNAP-MS. The relative abundance/intensity for each annotated feature is shown, and boxes colored green indicate the detection of a feature in a particular isolate.

Table S4: Annotations for putatively linked chemical features and the *apn* GCF. Annotations include the predicted structure class, select molecular fingerprints, and structure predictions and probability as computed through Sirius.

Table S5: Annotations for putatively linked chemical features and the PmNT3 GCF. Annotations include the predicted structure class, select molecular fingerprints, and structure predictions and probability as computed through Sirius.

Table S6: Putatively identified *cylC* homologs from *Microcystis* genomes containing the *PmNT3* gene cluster.


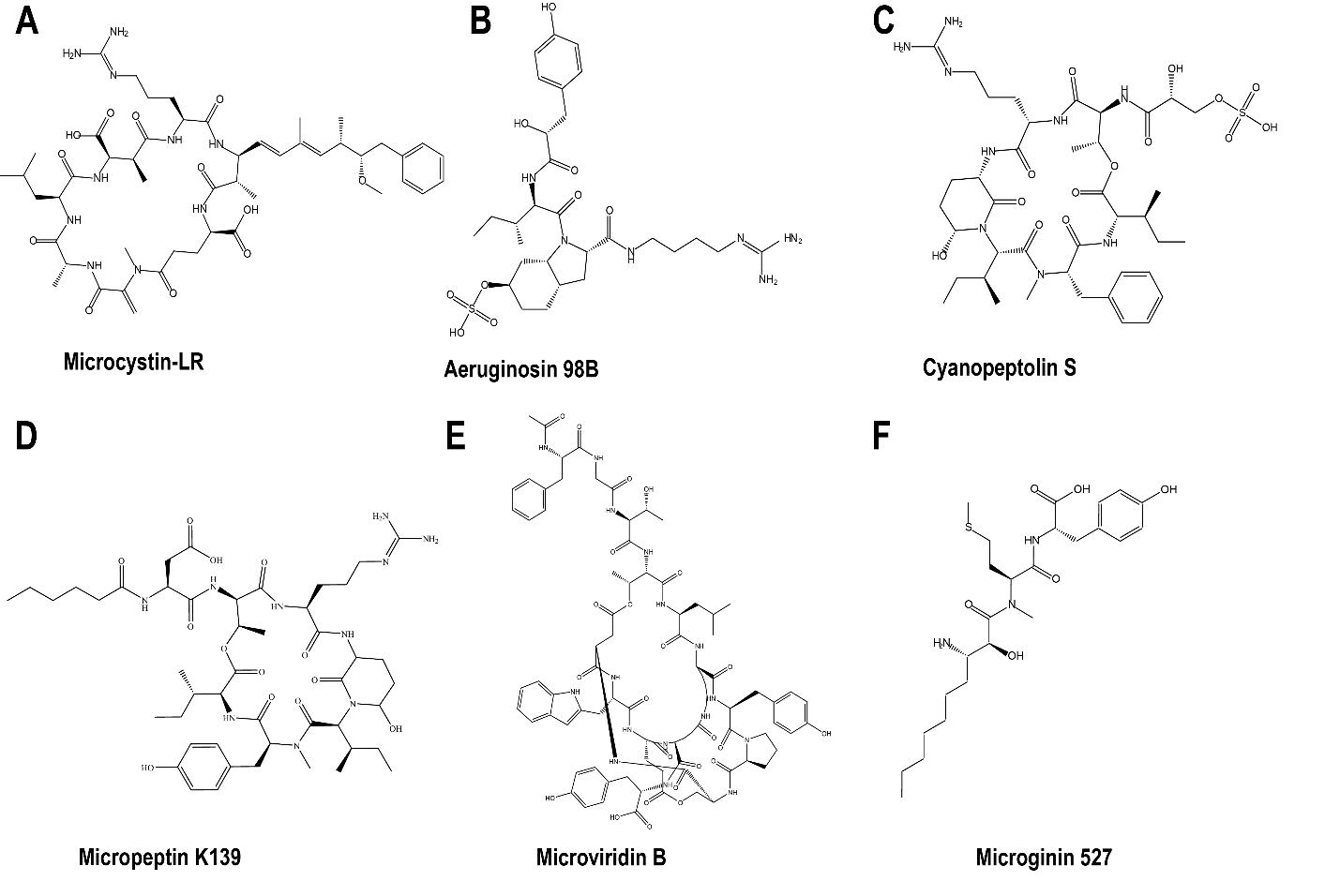


Figure S1: Examples of secondary metabolites known to be produced by *Microcystis* spp. Select congeners are shown, although the majority of Microcystis secondary metabolite classes are made up of several diverse congeners. Shown congeners include A) Microcystin-LR, B) Aeruginosin 98B, C) Cyanopeptolin S, D) Micropeptin K139, E) Microviridin B, and F) Microginin 527.


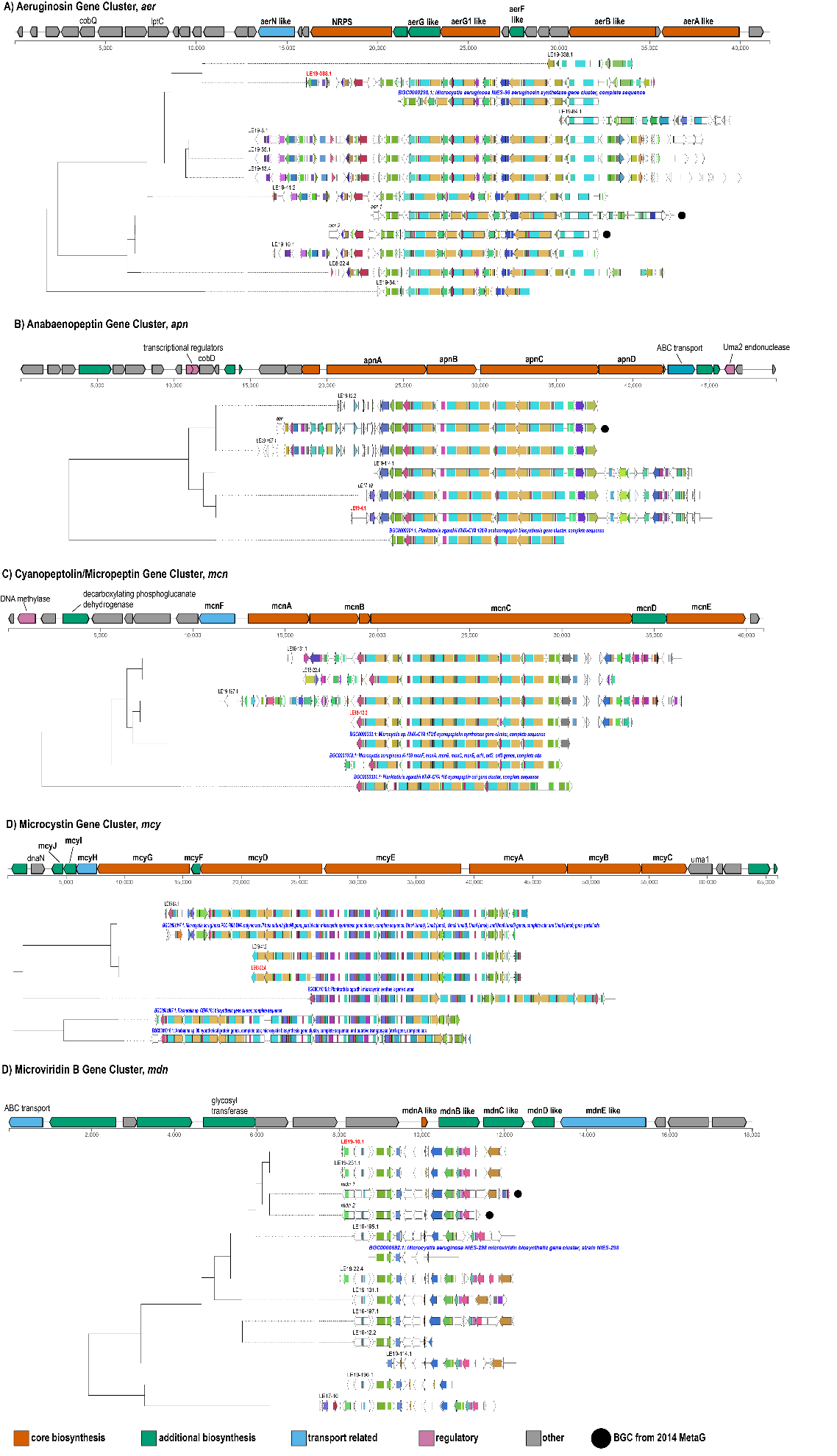


Figure S2: Gene schematics and Protein Family Database (PFAM) comparisons for similarly grouped and known BGCs from the WLE Culture Collection. The gene schematic at the top of each panel represents the genes within the GCF and annotations of note. Below each gene cluster are dendrograms based on the similarity of gene clusters and their PFAM annotations. These GCFs are known to encode characterized compounds including a) aeruginosin, b) cyanopeptolin or micropeptin, c) anabaenoepeptin, and d) microviridin B. MiBIG hits are included in PFAM comparisons highlighted in red.


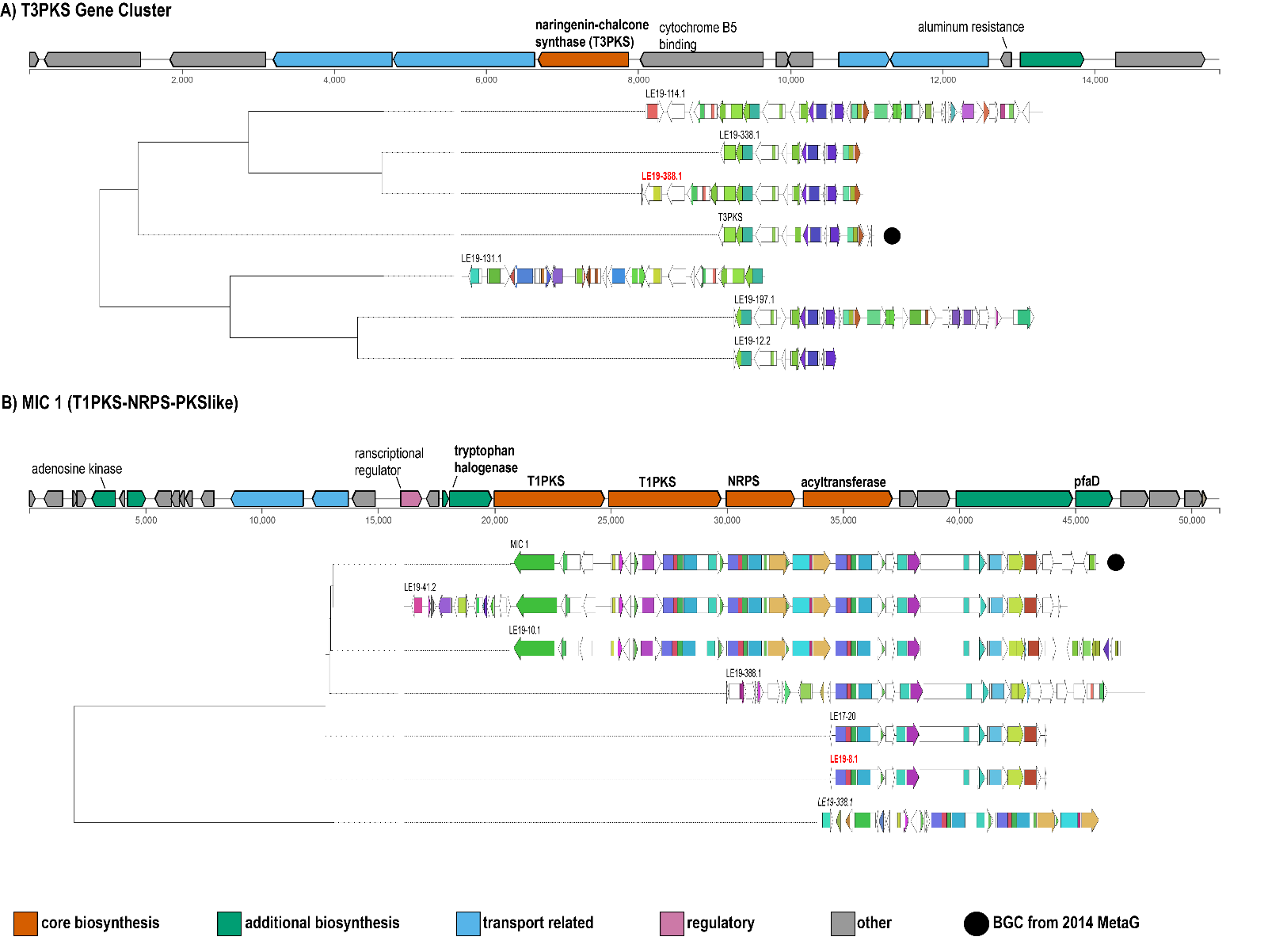


Figure S3: Gene Schematics and PFAM comparisons for similarly grouped and uncharacterized BGCs from the WLE Culture Collection. The gene schematic at the top of each panel represents the genes within the GCF and annotations of note. Below each gene cluster are dendrograms based on the similarity of gene clusters and their PFAM annotations. Clusters selected for analysis include A) T3PKS with a naringenin-chalcone synthase, and B) T1PKS-NRPS-PKSlike cluster that contains a tryptophan halogenase.


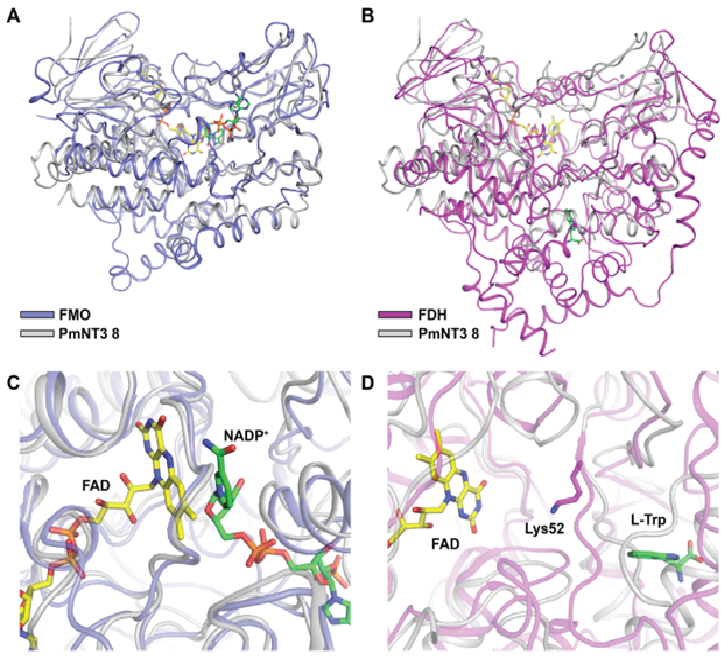


Figure S4. Comparison of PmNT3 8 homology model with FMO and FDH. (A) SWISS-MODEL homology model of PmNT3 8 (AF-A0A552JBG4-F1, 99.3% sequence identity, gray) superposed with the closest structural homologue (DALI) ancestral flavin monooxygenase AncFMO5 (PDB: 6SEK, blue) with RMSD of 2.1 Å^2^ . (B) PmNT3 8 homology model (gray) overlaid with closest FDH structural homologue, AetF (PDB: 8CJF, magenta) with RMSD of 3.3 Å^2^. (C) Active site view of AncFMO5 highlighting bound FAD (yellow) and NADP + (green). Binding sites for both cofactors are maintained in PmNT3 8. (D) Active site view of tryptophan halogenase AetF highlighting the conserved active site residue Lys52 and bound tryptophan.


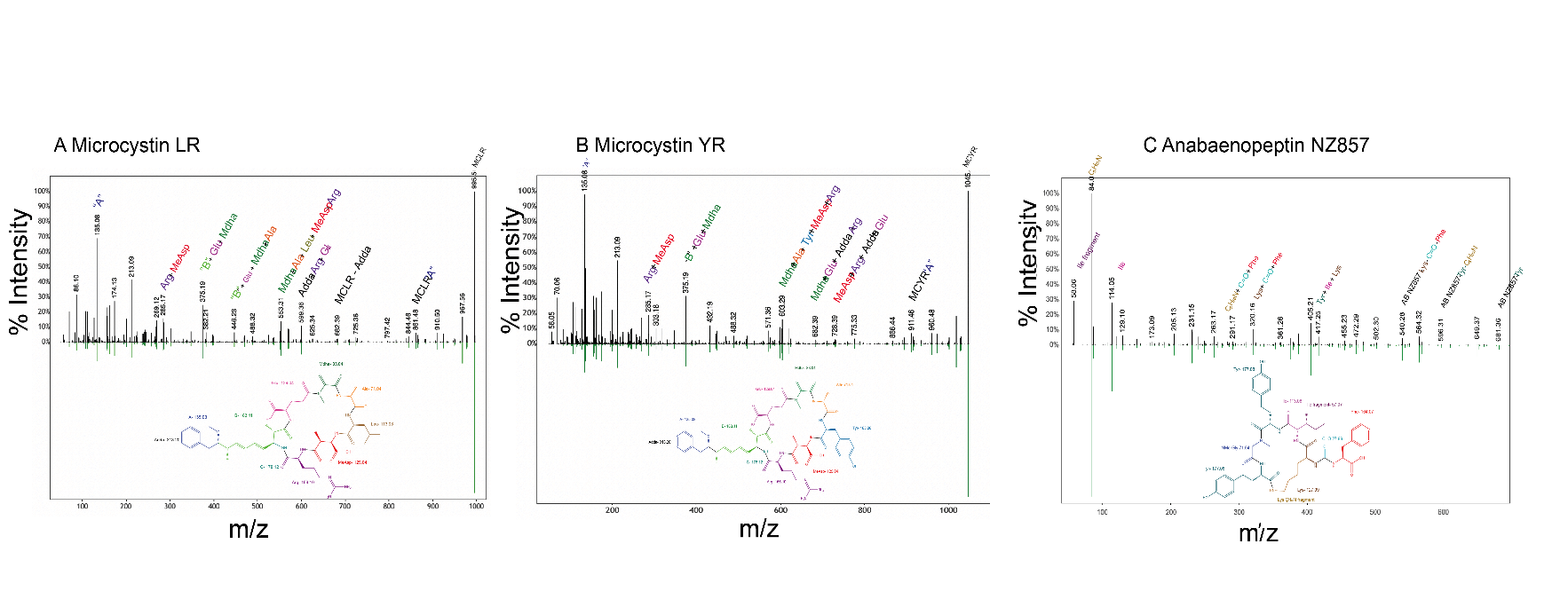
Figure S5: Mirror reference plots for select identified metabolites. The upper portion of MS/MS (black) is experimentally detected spectra, while the bottom portion (green) is database spectra. Mirror plots were generated via GNPS and spectral data provided in the GNPS library web server. A) Structure and MS/MS [M + H]^+^ annotation of Microcystin LR found. Adda fragment is 3-amino-9-methoxy-2,68-trimethyl-10-phenyl-4,6-decadienoic acid, and consists of fragments A, B, and C shown in the molecule; Mdha is *N*-methyldehydroalanine; MeAsp is β-methyl aspartic acid. B) Structure and MS/MS [M + H]^+^ annotation of Microcystin YR. Adda fragment is 3-amino-9-methoxy-2,68-trimethyl-10-phenyl-4,6-decadienoic acid, and consists of fragments A, B, and C, shown in the molecule; Mdha is *N*-methyldehydroalanine; MeAsp is β-methyl aspartic acid. C) Structure and MS/MS [M + H]^+^ annotation of Anabaenopeptin NZ857.. *N*Me-Gly is fragment is *N*-methylglycine.


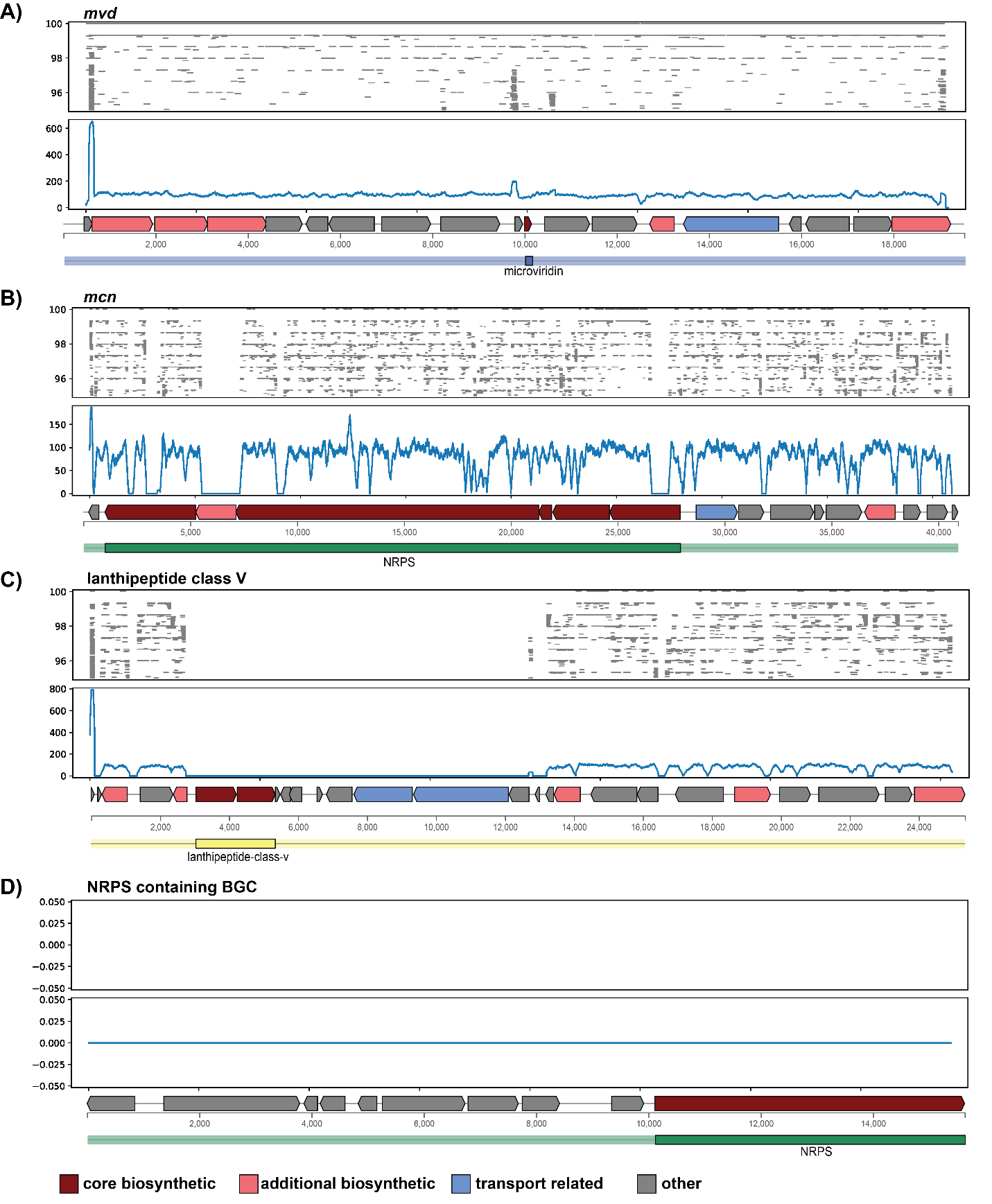


Figure S6: Examples of read mapping plots to determine the presence of ambiguously identified BGCs in WLE *Microcystis* Isolates. A) Complete coverage across the *mvd* gene cluster indicating it’s presence. B) Near complete coverage across the *mcn* gene cluster, indicating it’s presence with a minor deletion of a putative additional biosynthesis gene. C) Partial coverage of a lanthipeptide class V BGC, where core biosynthesis genes are absent. This gene cluster is considered absent. D) No coverage of a cryptic NRPS gene cluster, indicating its absence from the *Microcystis* isolate.
